# StackPR is a new computational approach for large-scale identification of progesterone receptor antagonists using the stacking strategy

**DOI:** 10.1038/s41598-022-20143-5

**Published:** 2022-09-30

**Authors:** Nalini Schaduangrat, Nuttapat Anuwongcharoen, Mohammad Ali Moni, Pietro Lio’, Phasit Charoenkwan, Watshara Shoombuatong

**Affiliations:** 1 Center of Data Mining and Biomedical Informatics, Faculty of Medical Technology, Mahidol University, Bangkok, 10700 Thailand; 2 Artificial Intelligence & Digital Health Data Science, School of Health and Rehabilitation Sciences, Faculty of Health and Behavioural Sciences, The University of Queensland, St Lucia, QLD 4072 Australia; 3 Department of Computer Science and Technology, University of Cambridge, Cambridge, CB3 0FD UK; 4 Modern Management and Information Technology, College of Arts, Media and Technology, Chiang Mai University, Chiang Mai, 50200 Thailand

**Keywords:** Data mining, Machine learning, Cheminformatics

## Abstract

Progesterone receptors (PRs) are implicated in various cancers since their presence/absence can determine clinical outcomes. The overstimulation of progesterone can facilitate oncogenesis and thus, its modulation through PR inhibition is urgently needed. To address this issue, a novel stacked ensemble learning approach (termed StackPR) is presented for fast, accurate, and large-scale identification of PR antagonists using only SMILES notation without the need for 3D structural information. We employed six popular machine learning (ML) algorithms (i.e., logistic regression, partial least squares, k-nearest neighbor, support vector machine, extremely randomized trees, and random forest) coupled with twelve conventional molecular descriptors to create 72 baseline models. Then, a genetic algorithm in conjunction with the self-assessment-report approach was utilized to determine *m* out of the 72 baseline models as means of developing the final meta-predictor using the stacking strategy and tenfold cross-validation test. Experimental results on the independent test dataset show that StackPR achieved impressive predictive performance with an accuracy of 0.966 and Matthew’s coefficient correlation of 0.925. In addition, analysis based on the SHapley Additive exPlanation algorithm and molecular docking indicates that aliphatic hydrocarbons and nitrogen-containing substructures were the most important features for having PR antagonist activity. Finally, we implemented an online webserver using StackPR, which is freely accessible at http://pmlabstack.pythonanywhere.com/StackPR. StackPR is anticipated to be a powerful computational tool for the large-scale identification of unknown PR antagonist candidates for follow-up experimental validation.

## Introduction

Progesterone receptor (PR) has emerged as a potential therapeutic target due to its increasingly recognized role in cancer development and progression. PR is implicated in various cancers such as breast and gynecological cancers (i.e., endometrial and ovarian cancers). However, their most studied implications lie in breast cancer. Cancer is still a major public health concern worldwide, with breast cancer ranking as the most commonly occurring in women. As of 2020, an estimated 2.3 million women were diagnosed with breast cancer resulting in 685,000 deaths globally^1^. In addition, ovarian and endometrial cancers ranked sixth and eighth out of the top ten cancers in women with incidence rates of 313,959 and 417,367, respectively^2^. Furthermore, according to the presence or absence of Estrogen receptor (ER) and PR, as well as human epidermal growth factor receptor 2 (Her2), breast cancer can be defined into 3 major groups. These include ER/PR-positive/Her2-negative tumors occurring in approximately 70% of patients, Her2 positive tumors in 15–20% of patients, and triple-negative tumors that lack all receptors in 15% of patients^3^.

PR belongs to the steroid nuclear receptor family which consists of other members such as ER, androgen receptor (AR), glucocorticoid receptor (GR), and mineralocorticoid receptor (MR). The role of PR in breast cancer through ER modulation has been investigated and as such, the expression of PR can estimate breast cancer prognosis^4,5^. Progesterone, the female sex hormone and endogenous ligand for PR, is involved in the development of the mammary glands, in the maintenance of pregnancy, and in the female menstrual cycle^6,7^. In the last decade, extensive research using murine models has recognized the role of progesterone in the development of breast cancer^8^. Additionally, these studies indicate that progesterone along with estrogen is a significant hormone involved in mammary gland development^9^. Triggered by estrogen, progesterone stimulates the growth of murine mammary stem cells (MSC) and inherently promotes the regeneration of the mammary gland^10^. Subsequently, breast cancer risk is amplified due to the increase in MSC numbers in response to progesterone^11,12^. This facilitates oncogenesis through the accumulation of replication mutations^13,14^. Therefore, modulation of progesterone through PR inhibition is vital for decreasing MSC and thus, slowing accumulation.

As stated above, breast cancer cells need estrogen and progesterone to grow hence, creating ER/PR positive or negative tumors. Determining the hormone response status of a tumor is the gold standard method and is crucial for patients to suitably benefit from endocrine therapy. Endocrine therapy used for hormone-positive breast cancer patients constitutes an easy-to-approach option for patients as it is generally well tolerated. However, A cohort study conducted on patients with breast cancer indicated that those with ER-positive/PR-negative tumors had a lower survival rate than those with ER-positive/PR-positive tumors^15,16^. This study suggests that, despite being ER-positive, the loss of PR is associated with the development of resistance in such tumors^17^. Thus, the research and development of PR-targeted compounds offer a great opportunity.

The lack of specificity regarding PR-targeting drugs exhibits a significant barrier in the clinical translation of PR-targeted therapies. Until now, the use of steroidal PR agonists has been mainly in the areas of oral contraception and postmenopausal hormone therapy^18–20^. However, the in vitro cell growth inhibitory effects afforded by PR antagonists particularly in ovarian, breast, prostate, and bone cancer cells, are now garnering attention as a potential anti-cancer regimen^21–25^. Furthermore, despite high in vitro antagonist activity against PR, recent clinical trials with mifepristone, a selective progesterone receptor modulator (SPRM), have largely been unsuccessful for ovarian cancer^23,26^. Despite achieving substantial activity to counter breast cancer in clinical trials, SPRMs have not been well endured by most participants due to their great affinity and responsiveness to GR^24^. Recently, interest in SPRMs for the treatment of breast cancer has been revived due to the discovery of potent progesterone antagonists having minimal antiglucocorticoid activity, such as telapristone acetate (TPA) and ulipristal acetate (UPA). TPA and UPA are currently in phase II and phase III clinical trials for treating endometriosis and breast cancer, and abnormal uterine bleeding, respectively^19,27–29^. Vilaprisan is another SPRM that underwent efficacy and safety assessment in phase III clinical trials of the oral treatment for uterine fibroids^30^. However, reports of hepato-cytotoxicity by SPRMs in other clinical trials halted the aforementioned one^31^. Similarly, concerns regarding hepato-cytotoxicity have limited the advancement of onipristone, a full progesterone antagonist, despite promising activity against breast and gynecological cancers. The clinical trial with onipristone is currently being re-evaluated with a lower drug dosage^32^. In this regard, there appears to be particular promise in developing newer PR antagonists.

The conventional process of novel drug discovery represents a costly, labor-, and time-intensive venture. These days, the use of computer-aided drug discovery methods has increasingly played essential and fundamental roles as part of the drug discovery process to alleviate the burden of labor and expense. As such, over the past decade, several computational approaches (i.e., molecular docking, quantitative structure–activity relationship (QSAR), and deep-learning) have been utilized for the exploration of PR modulators pertaining to 3D QSAR and docking studies of steroidal^33^ and non-steroidal^34–37^ analogs and, more recently, deep learning-based QSAR^38^. However, all these approaches might be limited in the quick and accurate identification of new PR antagonists from large-scale uncharacterized compounds. On the other hand, machine learning (ML) methods can utilize only SMILES information without the need for 3D structural information, highlighting their great efficiency in the large-scale identification of compounds.

Thus, in this study, we developed StackPR, a novel and stacked computational model for accurate and large-scale identification of compounds against PR by using only SMILES notation without the use of 3D structural information. Firstly, we comprehensively investigated the impact of different molecular descriptors and ML algorithms in the identification of PR antagonists by utilizing six popular ML algorithms (i.e., logistic regression (LR), partial least squares (PLS), k-nearest neighbor (KNN), support vector machine (SVM), extremely randomized trees (ET) and random forest (RF)) coupled with twelve conventional molecular descriptors. Secondly, a total of 72 different classifiers were developed and considered as baseline models. Then, a genetic algorithm cooperating with the self-assessment-report approach (GA-SAR) algorithm was employed to determine *m* out of the 72 baseline models for constructing the optimal meta-predictor using the stacking strategy. Thirdly, the SHapley Additive exPlanations (SHAP) algorithm was used to indicate the most important features for StackPR. Finally, molecular docking was conducted to determine the top compounds having a high binding affinity towards PR. The top-scoring compound was then further analyzed for its binding interactions and substructures.

## Materials and methods

### Dataset construction

The dataset used in this study was collected from the ChEMBL database (Target ID: CHEMBL208; version 25)^39^, which consisted of an initial set of 5,240 compounds with activity for PR. After the data curation process, a final data set of 1,168 compounds were obtained using IC_50_ (half-maximal inhibitory concentration) as the bioactivity unit for further investigation. IC_50_ denotes the amount of drug required for the inhibition of a biological process by half, thus stipulating the potency measure of an antagonist drug in pharmacological research^40^. Furthermore, Beck et al.^41^ reports that IC_50_ can be used effectively in building robust and reliable models to support SAR (structure–activity relationship) studies in drug discovery. Bioactivity thresholds of IC_50_ ≤ 1 μM and ≥ 10 μM were applied to the final data set of 1,168 to separate compounds into active and inactive groups, respectively. Compounds with biological activity IC_50_ values ranging between 1 and 10 μM (i.e., intermediate compounds), which consisted of 445 compounds, were not selected for this study. As a result, a high-quality dataset was obtained which consisted of 463 active (positive samples) and 260 (negative samples) inactive compounds. Of these compounds, 370 active and 208 inactive compounds are considered as the training dataset (called TRN515), and the remaining compounds (93 active and 52 inactive compounds) are used as the independent test dataset (called IND145).

### Feature engineering

Molecular fingerprints stipulate data regarding the substructures that are inherently present in a molecule and thus, are important in QSAR studies. Using the built-in function of the PADEL-descriptor software, salt was removed and tautomers were standardized during structure pre-processing. Herein, the SMILES notation was used as the input for descriptor calculations. SMILES strings are a useful representation of molecules while gaining the advantages in terms of their storage and handling^42^. We utilized twelve molecular fingerprints, to generate structural features of the investigated compounds. The twelve fingerprints consisted of AP2D, Circle, CKD, CKDExt, CKDGraph, Estate, FP4, FP4C, Hybrid, KR, MACCS, and PubChem. Details of each fingerprint descriptor can be found in Table 1. Herein, all molecular descriptors can be extracted using the Python environment^43^.

**Table 1:** Summary of twelve molecular fingerprints used in this study.

Fingerprint	Abbreviation	#Feature	Description	Ref
2D atom pair	AP2D	780	Presence of atom pairs at various topological distances	^87^
CDK	CKD	1024	Fingerprint of length 1024 and search depth of 8	^88^
CDK extended	CKDExt	1024	Extends the fingerprint with additional bits describing ring features	^88^
CDK graph only	CKDGraph	1024	A special version that considers only the connectivity and not bond order	^88^
Circle	Circle	1024	Circular fingerprint	^89^
EState	EState	79	Electrotopological state atom types	^90^
Hybrid	Hybrid	1024	CDK hybridization fingerprint	^89^
Klekota–Roth	KR	4860	Presence of chemical substructures	^91^
MACCS	MACCS	166	Binary representation of chemical features defined by MACCS keys	^92^
Pubchem	Pubchem	881	Binary representation of substructures defined by PubChem	^93^
Substructure	FP4	307	Presence of SMARTS patterns for functional groups	^94^
Substructure count	FP4C	307	Count of SMARTS patterns for functional groups	^94^

### StackPR framework

**Figure 1:**
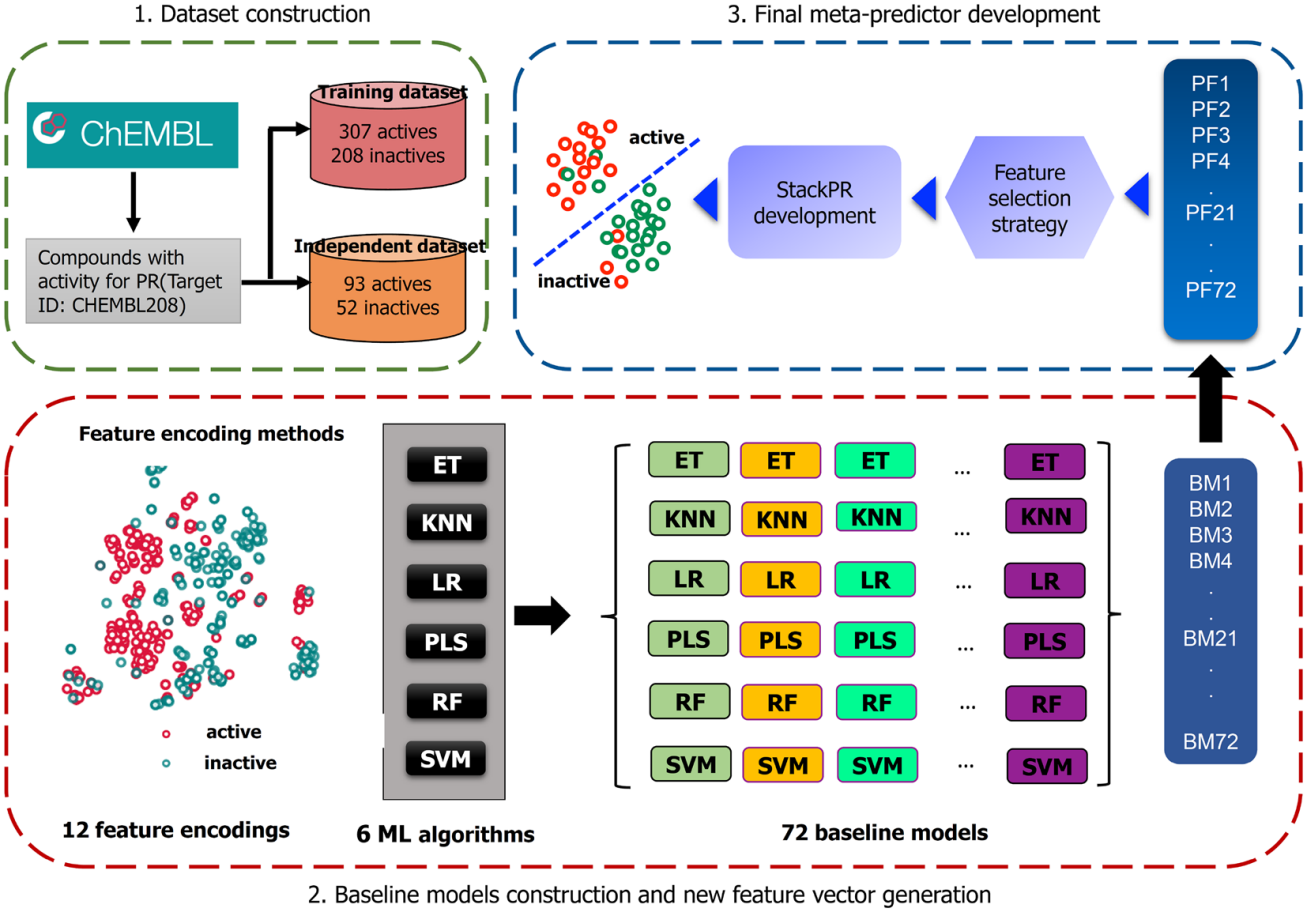
System flowchart of the proposed StackPR. The overall workflow for the development of StackPR contains three major steps: dataset construction, baseline model construction and new feature vector generation and meta-predictor development.

In this section, we describe our stacked ensemble learning framework (named StackPR) designed for the high-throughput identification of PR antagonists. Unlike other conventional ensemble strategies, the stacking strategy integrates the strengths of different predictive models without human intervention to generate the final meta-predictor^44–47^. To date, numerous previous studies have indicated that the final meta-predictor can potentially attain a more stable predictive performance^48–50^. The overall workflow for the development of StackPR contains three major steps (i.e., baseline model construction, new feature vector generation, and meta-predictor development) as provided in the paragraphs hereafter (Fig. 1).

In the first step, we used twelve different molecular descriptors (AP2D, Circle, CKD, CKDExt, CKDGraph, Estate, FP4, FP4C, Hybrid, KR, MACCS, and PubChem) in combination with six ML algorithms (LR, PLS, KNN, SVM, ET, and RF) to generate a collection of baseline models. Specially, all the molecular descriptors were normalized in the range of 0–1, when training the six ML classifiers. As a result, a total of 72 baseline models were created. Herein, we utilized ET, LR, RF, and SVM classifiers with their optimal parameters, where the search range is recorded in Table 2. In the meanwhile, KNN and PLS classifiers were generated by using their default parameters. All the baseline models were built and optimized using the Scikit-learn v0.22.0 package^51^ with a tenfold cross-validation test. In addition, we evaluated and analyzed the effect of molecular descriptors and ML classifiers in PR antagonist identification by performing a tenfold cross-validation test to determine the best-performing baseline model in terms of Matthew’s coefficient correlation (MCC). In addition, we evaluated and analyzed the effect of molecular descriptors and ML classifiers in PR antagonist identification by performing a tenfold cross-validation test to determine the best-performing baseline model in terms of Matthew’s coefficient correlation (MCC).

**Table 2:** Hyperparameter search details for six different ML classifiers.

Method	Parameters	Range of parameters
ET	n_estimators	[20, 50, 100, 200, 500]
KNN	Number of neighbours	Default
LR	C	[0.001, 0.01, 0.1, 1, 10, 100]
PLS	#Components	Default
RF	n_estimators	[20, 50, 100, 200, 500]
SVM	C	[1, 2, 4, 8, 16, 32]

Columns 2 and 3 represents the parameter name used in the Scikit-learn library and the range of parameter used to develop the model, respectively.

In the second step, all the 72 baseline models were utilized for constructing a new feature vector. Each baseline model can provide the information on the predicted confidence of being PR antagonists. Herein, the predicted confidence is treated as a probabilistic feature (PF), where PF is in the range of 0–1. For a given compound *C*, its feature vector is created by concatenating all PFs generated by all the 72 baseline models, which can be defined by:

1 $$\mathrm{nFeat}(C)=\{{PF}_{BM\left(1\right)},{PF}_{BM\left(2\right)},{PF}_{BM\left(3\right)},\dots ,{PF}_{BM\left(72\right)}\}$$

where ${PF}_{BM\left(i\right)}$ is the *i*th PF derived from the *i*th baseline model ($BM(i)$) of compound *C.* Thus, $\mathrm{nFeat}(C)$ is the 72-D feature vector and considered as a new feature vector.

In the last step, a meta-predictor was built by using the 72-D feature vector coupled with the RF algorithm (called mRF model). To improve the discriminative ability of the new feature vector, in this study, we also utilized the GA-SAR algorithm^52^ to determine *m* out of the 72 PFs, where the number of *m* was set within the range of 5–20 with an interval of 1. Specifically, the chromosome of the GA-SAR algorithm contains two main genes, binary (GA-gene) and parametric (GA-chrom). The binary (GA-gene) and parametric (GA-chrom) are the dominant genes of the chromosome of the GA-SAR algorithm, which are used for feature and parameter optimizations, respectively. For the mRF model, the chromosome consists of *n* = 72 binary genes (*bg*_i_) for selecting *m* important PFs and 3-bit genes for optimizing the parameters of mRF (n_estimators $\in$ {20, 50, 100, 200, 500}). If *bg*_*i*_ = 1, the *i*th feature is selected to construct the mRF model; otherwise, the *i*^*th*^ feature is excluded from the optimal feature set.

### Performance evaluation

The performance evaluation results of StackPR and its baseline models were examined in terms of five well-known performance metrics, including MCC, *F*-value, sensitivity (Sn) and specificity (Sp), and accuracy (ACC)^53–55^ as described follows:

2 $$\mathrm{MCC}=\frac{\mathrm{TP}\times \mathrm{TN}-\mathrm{FP}\times \mathrm{FN}}{\sqrt[]{(\mathrm{TP}+\mathrm{FP})(\mathrm{TP}+\mathrm{FN})(\mathrm{TN}+\mathrm{FP})(\mathrm{TN}+\mathrm{FN})}}$$

3 $$\mathrm{F}-\mathrm{value}=2\times \frac{\mathrm{TP}}{2\mathrm{TP}+\mathrm{FP}+\mathrm{FN}}$$

4 $$\mathrm{Sn}=\frac{\mathrm{TP}}{\left(\mathrm{TP}+\mathrm{FN}\right)}$$

5 $$\mathrm{Sp}=\frac{\mathrm{ TN}}{\left(\mathrm{TN}+\mathrm{FP}\right)}$$

6 $$\mathrm{ACC}=\frac{\mathrm{TP}+\mathrm{TN}}{\left(\mathrm{TP}+\mathrm{TN}+\mathrm{FP}+\mathrm{FN}\right)}$$

where TP and TN indicate the number of true positives and true negatives, respectively. Moreover, FP and FN represent the number of false positives and false negatives, respectively^56–61^.

### Molecular docking

Molecular docking was performed as a virtual screening to identify potential inhibitors for human progesterone receptors. In this study, 1168 bioactive compounds with known bioactivity described using IC_50_, were collected from the ChEMBL database^39^ and preprocessed as input ligands for investigation. The molecular structure of the ligands was generated and optimized to achieve low-energy conformers using the OpenBabel software^62^. The co-crystal structure of the human progesterone receptor in complex with asoprisnil (PDB ID: 2OVH) was retrieved from the Protein Data Bank^63^ to be used as a receptor molecule for virtual screening. The protein structure was preprocessed by removing water molecules and adding Gasteiger charges and missing hydrogen atoms. The protein structure was additionally cleaned up by repairing bonds and removing non-polar hydrogens and lone pair atoms using MGLTools^64^. Consequently, grid boxes with the dimensions of 40 × 40 × 60 Angstrom were applied to the center of ligands inside the binding cavity of the protein receptor. Parameters for molecular docking were defined by default with a seed number of 1000 using Autodock Vina^65,66^. The method was validated using ligand re-binding approaches and the calculated RMSD of the atomic position between co-crystallized ligand and re-binding ligand was observed to be 0.322 Angstroms which is acceptable for further investigations. The binding energy (Kcal/mol) was calculated during virtual screening using the built-in scoring function of Autodock Vina and the compounds exhibiting the lowest binding energy were chosen for further investigation. The docked poses and binding interactions were visualized using the PyMOL Molecular Graphics System, version 2.2.3 (Schrödinger LLC, 2010).

## Results and discussion

### Analysis of applicability domain

Applicability domain (AD) analysis is key for reliable model predictions which can be utilized for all models^45,64,65^. Several approaches for AD analysis have been proposed however, herein the t-distributed stochastic neighbor embedding (t-SNE)^66,67^ was used for visual investigation of the feature distribution pertaining to the twelve molecular fingerprints. Supplementary Fig. S1A–L elucidates the 2D feature distribution space where the TRN515 and IND145 datasets are represented by red and green dots, respectively. In order for the compounds to fall within the AD of the model, compounds from the IND145 dataset must be present in the defined boundary of the TRN515 dataset. Conversely, compounds are considered outside the AD of the model if they fall beyond the established boundary stated above. From Supplementary Fig. S1, the chemical spaces for all twelve molecular fingerprints were observed to be overlapping for both the TRN515 and IND145 datasets. Thus, it can be inferred that the AD is well defined for these twelve molecular fingerprint-based models developed herein.

### Performance evaluation of different molecular descriptors and ML algorithms

**Figure 2:**
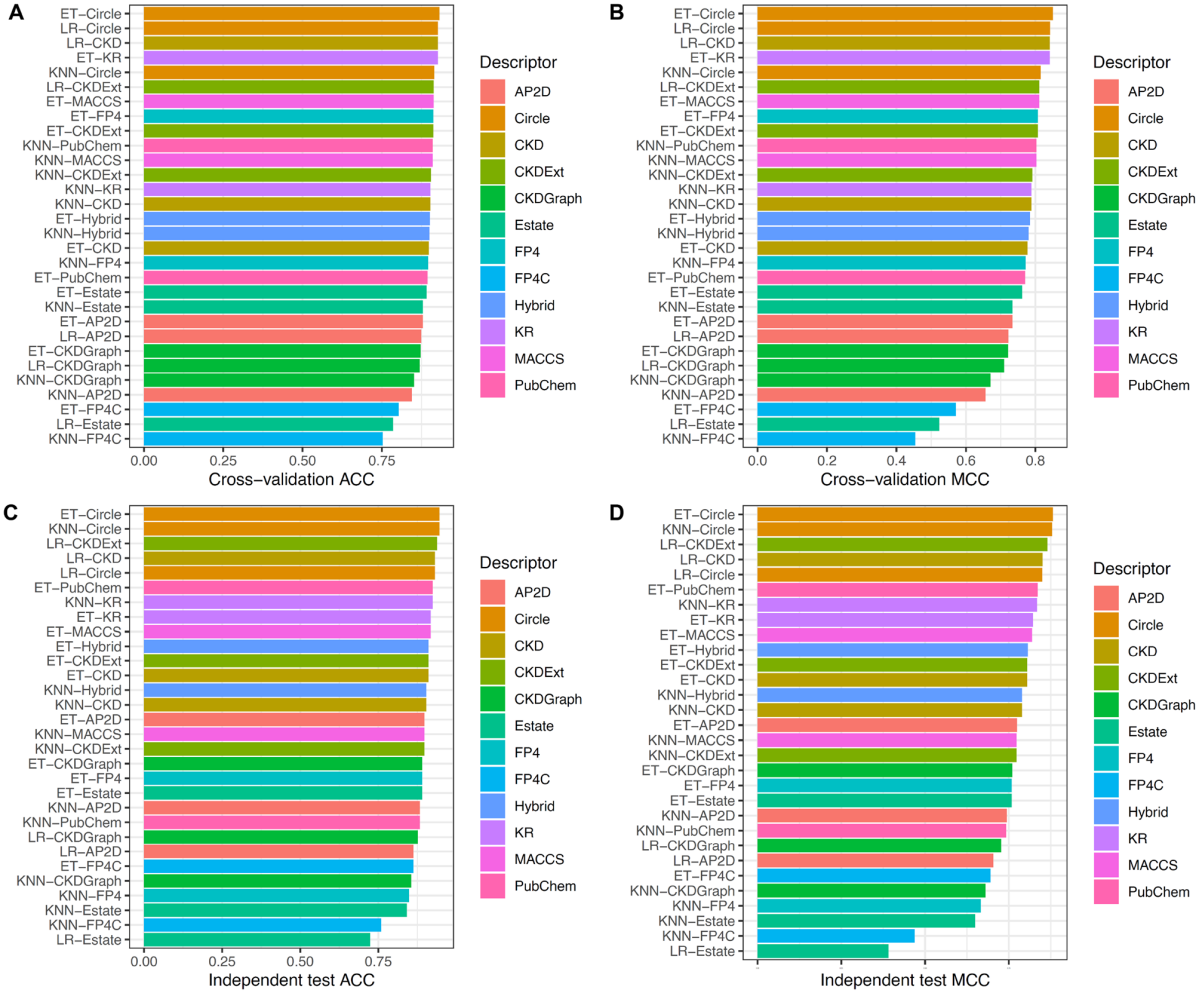
Performance evaluations of top 30 baseline models. (**A**, **B**) Cross-validation ACC and MCC of top 30 baseline models. (**C**, **D**) Independent test ACC and MCC of top 30 baseline models.

Here, we assessed the performance of several ML classifiers trained using utilizing twelve molecular descriptors and six ML algorithms. All the 72 classifiers (6 MLs × 12 descriptors) were assessed by using tenfold cross-validation and independent tests. The performance evaluation results of the 72 classifiers are provided in Fig. 2 and Supplementary Tables S1–S4. Note that the classifier with the highest MCC was deemed to be the best-performing classifier in this study. Supplementary Table S3 shows that the top-five molecular descriptors having the highest cross-validation performance were Circle, KR, CKD, CKDExt, and Hybrid with corresponding average ± standard deviation and MCC of 0.834 ± 0.018, 0.802 ± 0.023, 0.797 ± 0.024, 0.796 ± 0.014, and 0.784 ± 0.013, respectively. In the case of performance results of the six ML methods, Supplementary Table S4 shows that ET and RF achieve superior performance in terms of MCC with a range of 0.768–0.770. It could be noticed that the top-five classifiers having the highest cross-validation performance consisted of ET-Circle, RF-Circle, SVM-Circle, LR-Circle, and LR-CKD with corresponding MCC of 0.850, 0.846, 0.843, 0.842 and 0.841, respectively (Fig. 2 and Supplementary Table S1). Interestingly, four out of the top-five classifiers were developed using the Circle descriptor. This indicates that the Circle descriptor was beneficial for the identification of PR antagonists. For the performance on the IND145 dataset, the top-five classifiers yielded MCC with a range of 0.849–0.881. Although the best-performing classifier (ET-Circle) could perform well in the identification of PR antagonists, ensemble models that can automatically integrate the individual strengths of the above-mentioned classifiers are admissible^45,49,50,67^.

### Construction of StackPR

Although the ET-Circle model has yielded good performances, its overall prediction performance is still unsatisfactory for real therapeutic applications. Thus, we herein employed an ensemble approach that could take advantage of several ML-based classifiers to construct a stable stacked model. Specifically, we built two mRF models coupled with two types of new feature vectors, including the 72-D and m-D feature vectors (referred herein as the optimal model and control, respectively). The 72-D feature vector was obtained by all the 72 PFs, while the m-D feature vector was obtained from the GA-SAR algorithm. Table 3 provides the overall performance of the 72-D and m-D feature vectors. After performing the GA-SAR algorithm, we obtained the optimal feature set having m = 10 selected PFs derived from ten baseline models of KNN-AP2D, LR-CKDExt, SVM-CKDExt, ET-CKDExt, KNN-MACCS, RF-PubChem, SVM-KR, LR-FP4, RF-Circle, and PLS-Hybrid. As shown in Table 3, the 10-D feature vector achieves the overall best performance compared with the 72-D feature vector on both the TRN515 and IND145 datasets in approximately all performance metrics, except for AUC. Remarkably, ACC, Sn, and MCC of the 10-D feature vector were 3.45, 4.30, and 7.31% higher than the 72-D feature vector on the independent test dataset. Altogether, the mRF model in conjunction with the 10-D feature vector (called the optimal feature vector) is deemed to be the final model herein and referred to as StackPR for the convenience of discussion.

**Table 3:** Performance comparison of the optimal model and control on the training and independent test datasets.

Evaluation strategy	Method^a^	Dimension	ACC	Sn	Sp	MCC	AUC
Cross-validation	Control	72	0.927	0.946	0.894	0.843	0.972
	Optimal	10	0.950	0.970	0.913	0.893	0.976
Independent test	Control	72	0.931	0.935	0.923	0.852	0.984
	Optimal	10	0.966	0.978	0.942	0.925	0.978

^a^The optimal model and control are developed by using mRF models coupled with the 72-D and 10-D feature vectors, respectively.

### Stacking improves the prediction performance

**Figure 3:**
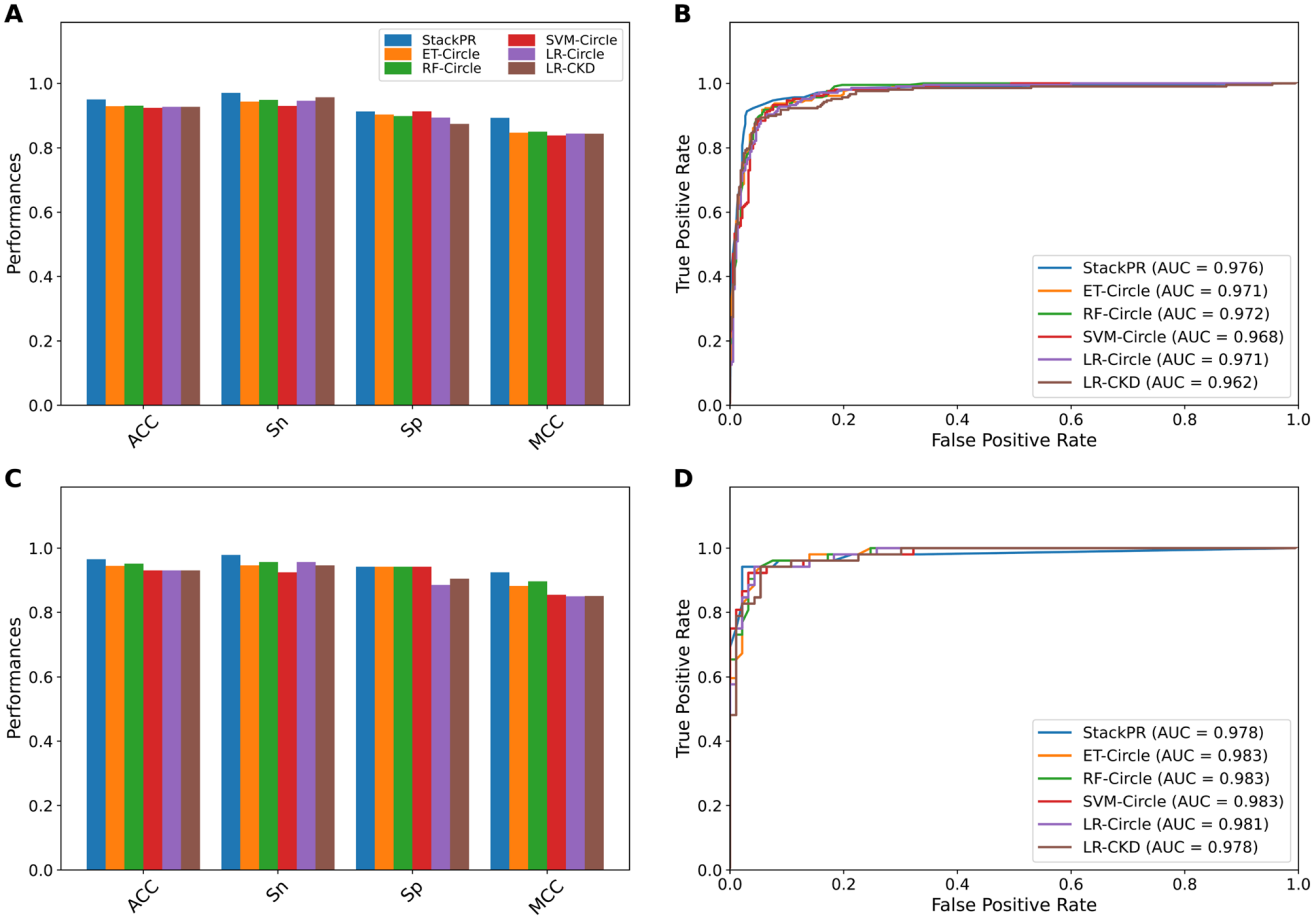
Performance comparison of StackPR with the top five baseline models on the Main-TRN (**A**, **B**) and Main-IND (**C**, **D**) datasets. Prediction results of StackPR with the top five baseline models in terms of MCC, Sn, Sp and MCC (**A**, **C**). ROC curves and AUC values of StackPR with the top five baseline models (**B**, **D**).

In this section, we aim to reveal the effectiveness of the stacking strategy by comparing the performance of StackPR with the top five baseline models as judged by the cross-validation MCC. These baseline models included ET-Circle, RF-Circle, SVM-Circle, LR-Circle, and LR-CKD. The detailed results of their performance evaluations are provided in Fig. 3, Table 4, and Supplementary Table S5. Table 4 shows that StackPR’s ACC, Sn, MCC, and *F*-value were better than the top five baseline models on both the TRN515 and IND145 datasets. Interestingly, ACC, Sn, and MCC of StackPR were 0.950, 0.970, 0.893, and 0.958, which were 1.90, 2.70, 4.31, and 1.52%, respectively, higher than the best-performing baseline model ET-Circle in the TRN515 dataset. StackPR also attained a better performance as compared with ET-Circle on the IND145 dataset. For the performance on the independent test dataset, StackPR’s ACC, Sn, and MCC were 0.966, 0.978, 0.925, and 0.973, respectively. To be specific, these three-performance metrics were higher than that of the ET-Circle model by 2.07, 3.23, 4.34, and 1.67%, respectively. However, StackPR provided a slightly lower AUC as compared to the ET-Circle model (0.978 versus 0.983), StackPR could identify more true compounds against PR (TP) in terms of both the TRN515 (299 versus 290) and IND145 (91 versus 88) datasets (Table 4 and Supplementary Table S5). These results indicated that the stacking approach used in StackPR effectively integrated the advantages of the baseline models, contributing to the improvement in performance.

**Table 4:** Performance comparison of StackPR and top five baseline models on the training and independent test datasets.

Evaluation strategy	Method	ACC	Sn	Sp	MCC	AUC	*F*-value
Cross-validation	ET-circle	0.931	0.943	0.909	0.850	0.976	0.943
	RF-circle	0.929	0.943	0.904	0.846	0.983	0.942
	SVM-circle	0.927	0.935	0.913	0.843	0.983	0.940
	LR-circle	0.927	0.946	0.894	0.842	0.983	0.939
	LR-CKD	0.927	0.957	0.875	0.841	0.981	0.939
	StackPR	0.950	0.970	0.913	0.893	0.976	0.958
Independent test	ET-circle	0.945	0.946	0.942	0.881	0.983	0.957
	RF-circle	0.945	0.946	0.942	0.881	0.983	0.957
	SVM-circle	0.938	0.946	0.923	0.866	0.983	0.951
	LR-circle	0.931	0.957	0.885	0.849	0.981	0.947
	LR-CKD	0.931	0.946	0.904	0.850	0.978	0.946
	StackPR	0.966	0.978	0.942	0.925	0.978	0.973

### Analysis of new feature vector

**Figure 4:**
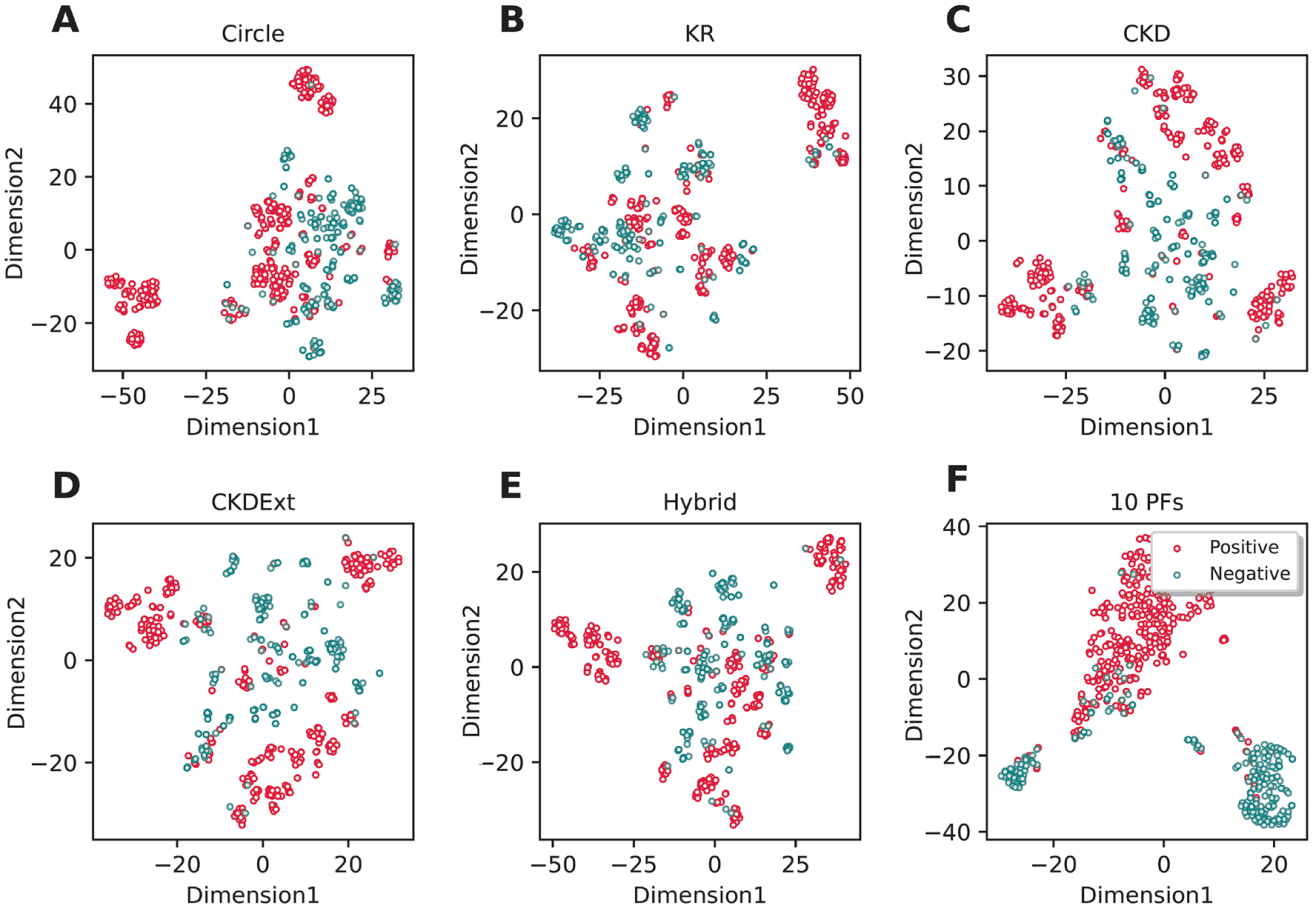
t-distributed stochastic neighbor embedding (t-SNE) distribution of positive and negative samples on the training dataset.

In this section, we investigated the performance of the optimal feature vector by testing and comparing its performance with the twelve conventional molecular descriptors. Supplementary Tables S6–S7 provide the predictive performance of the optimal feature vector against the twelve conventional molecular descriptors. For the sake of fairness, the RF classifier was employed to train different models coupled with the twelve molecular descriptors and build respective prediction models. For the convenience of the comparison purpose, we conducted the performance comparison of the optimal feature vector with only the top five molecular descriptors having the highest cross-validation MCC, including Circle, KR, MACCS, FP4, and CKDExt. We noticed that the top five beneficial molecular descriptors achieved the overall best performance as compared with the top five beneficial molecular descriptors on both the TRN515 and IND145 datasets as judged by ACC, Sn, and Sp (Supplementary Tables S6–S7). Remarkably, for the performance on the IND145 dataset, the optimal feature vector’s ACC, Sn, and MCC were 2.07–6.90, 2.15–4.3,0, and 4.34–15.18%, respectively, higher than that of the top five molecular descriptors. In addition, the 2D feature space of the optimal feature vector and the top five molecular descriptors were depicted by using the t-distributed stochastic neighbor embedding (t-SNE)^68,69^, where the red and green dots represent positive (active compounds) and negative (inactive compounds) samples, respectively (Fig. 4). Overall, we observe that the distributions of the five top-five beneficial molecular descriptors did not show a clear separation between the two classes (Fig. 4A–E). On the other hand, the optimal feature vector was able to provide a clear separation between the two classes (Fig. 4F). Altogether, these results indicate that our proposed 10-D feature vector derived from the combination of several molecular descriptors and ML algorithms had a more discriminative ability to capture the key information of active and inactive compounds against PR, contributing to the performance improvement.

### Mechanistic interpretation of StackPR

**Figure 5:**
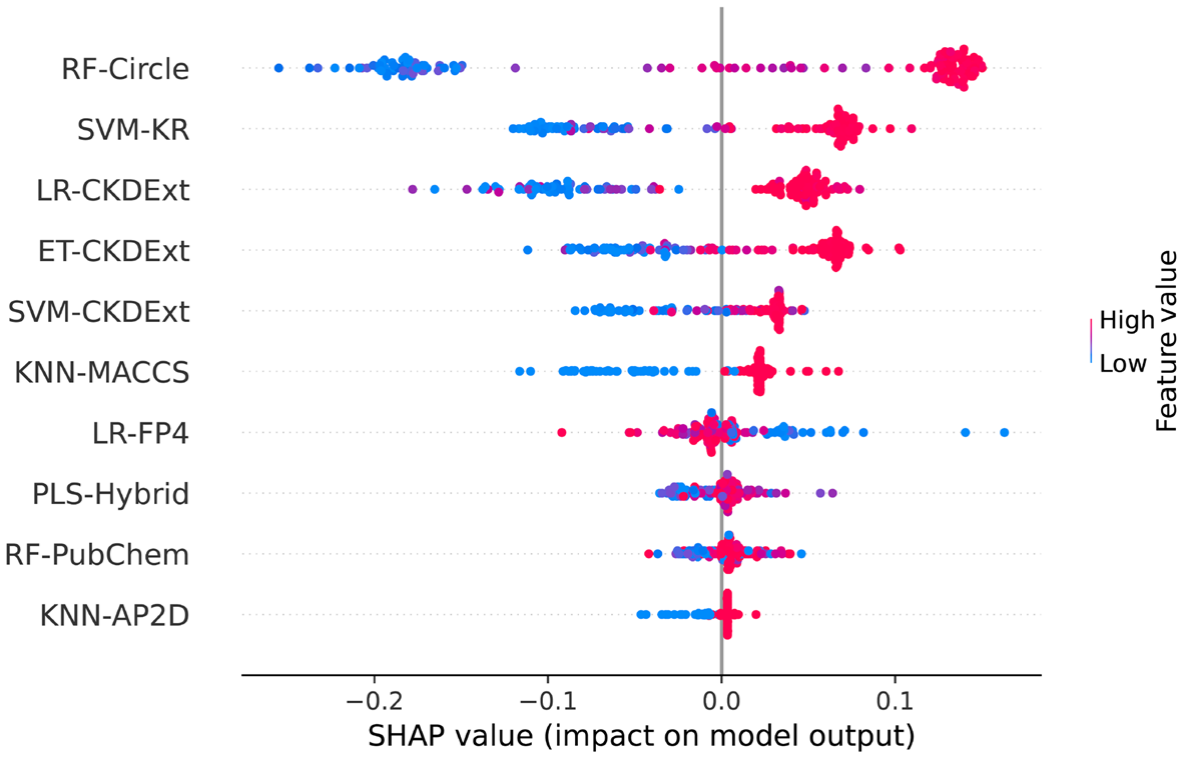
Feature importance from StackPR as ranked by SHAP values based on the training datasets. Such SHAP values represent the directionality of features where positive and negatives SHAP values influences the predictions toward positive and negative samples, respectively.

**Figure 6:**
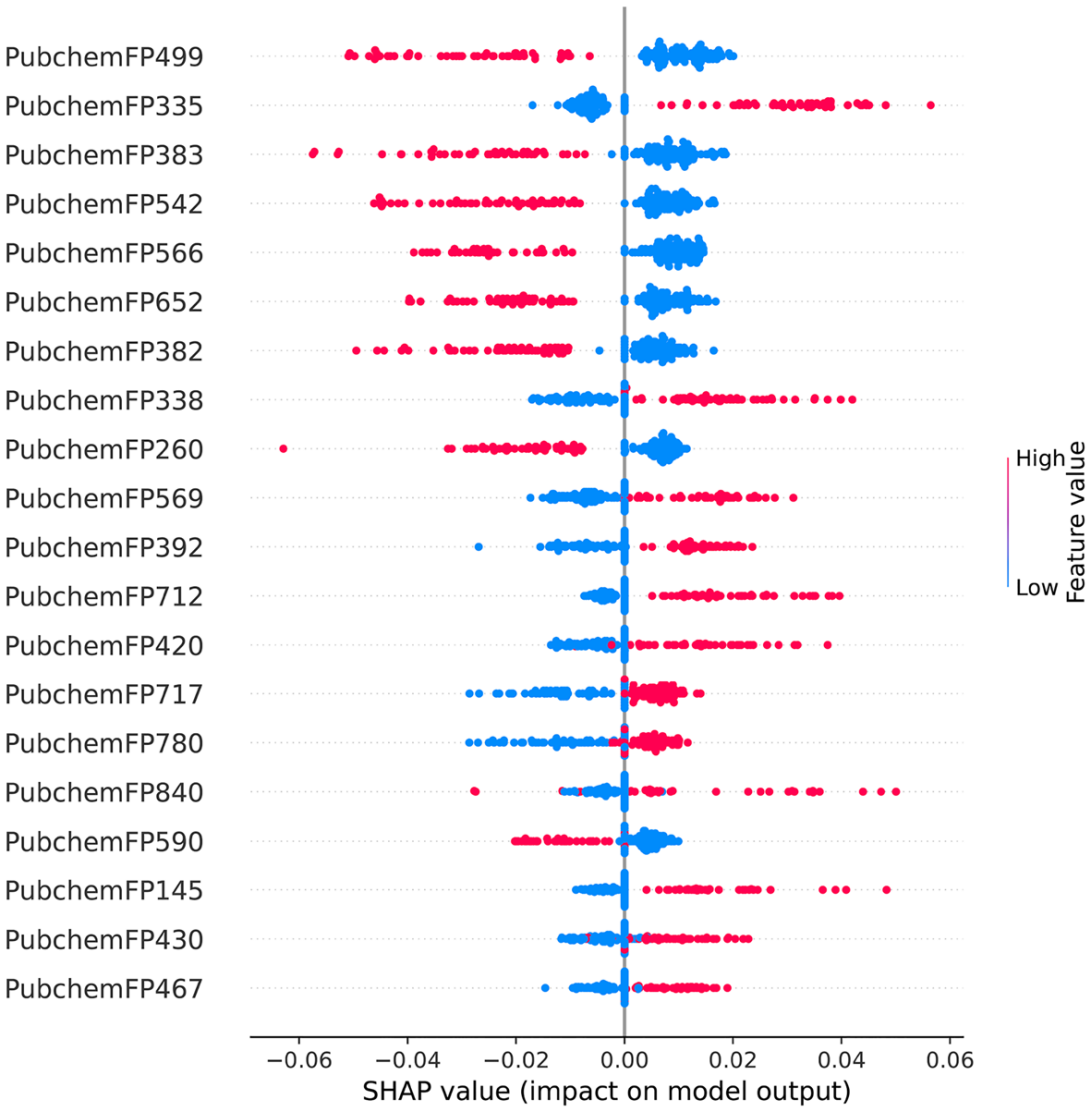
Feature importance from the RF-PubChem model as ranked by SHAP values based on the training datasets. Such SHAP values represent the directionality of features where positive and negatives SHAP values influences the predictions toward positive and negative samples, respectively.

To better understand the impact governing each feature of the StackPR model, an analysis of the feature importance was conducted. Herein, we utilized the SHAP framework^70^ to calculate the value of each feature while also shedding light on the output of the model. SHAP is a game-theoretic approach used to explain the output of any ML classifier and represents a crucial role in many bioinformatics applications. The positive and negative SHAP values indicate that the prediction model better favors active or inactive compounds, respectively. Figure 5 shows that the top five informative PFs based on SHAP values were derived from RF-Circle, SVM-KR, LR-CKDExt, ET-CKDExt, and SVM-CKDExt. Interesting, RF-Circle is found in both the top-performing classifier and the top-ranked informative PFs based on SHAP values. As can be seen in Fig. 6, the top-twenty most important informative features as deduced by SHAP values were PubChemFP499, PubChemFP335, PubChemF383, PubChemFP542 PubChemFP566, PubChemFP652, PubChemFP382, PubChemFP338, PubChemFP260, PubChemFP569, PubChemFP392, PubChemFP712, PubChemFP420, PubChemFP717, PubChemFP780, PubChemFP840, PubChemFP590, PubChemFP145, PubChemFP430, and PubChemFP467. In addition, as stated above, high SHAP value (positive scale) with high feature value (represented by red) which are most likely to have an impact on the substructure of the compounds were seen in twelve out of top-twenty informative features (Fig. 6 and Table 5) representing, five aliphatic hydrocarbons (i.e., PubChemFP335, PubChemFP712, PubChemFP717, PubChemFP780, PubChemFP430), five nitrogen-containing (i.e., PubChemFP338, PubChemFP569, PubChemFP392, PubChemFP145, PubChemFP467), one alcohol (i.e., PubChemFP840) and one carbonyl group (i.e., PubChemFP420; which is common to several classes of organic compounds such as aldehyde and ketone). Therefore, it can be inferred that aliphatic hydrocarbons and nitrogen-containing compounds represent substructures that have a high impact on PR antagonism. Further exploration into the PubChem substructure descriptions (Table 5), offers observations that bioisoteric transformations occur when the CH group of compounds containing heteroaromatic rings are substituted with a N atom (i.e., PubChemFP145). This substation exerts antagonistic effects by mirroring the binding of natural ligands^71^. Furthermore, the above-mentioned features belong mainly to N-methylmethanamine, ethylenediamine, isopropylamine etc. which are all precursors of many significant PR antagonists such as mifepristone, lonaprisan and vilaprisan. However, several steroidal SPRMs with a dimethylamino substituent have shown an increase in liver enzyme upon continued use^72^. Nevertheless, novel analogues of mifepristone with increased PR selectivity over GR has been identified^73^. As for the aliphatic substructures observed in Table 5, Nishiyama et al.^74^, identified that alkyl substitutions at 4-Alkyl analogs of phenanthridin-6(5H)-one skeleton show potent PR antagonistic activity. Additionally, the 1-propynyl substituent at the C-17 position of mifepristone accounts for its high PR binding affinity^75^. Similarly, Richardson et al.^76^, discovered that alkyl substituents on the N1 of the indole skeleton played an important role in the binding affinity of compounds to PR whereby changing the substituent from methyl to ethyl and then from ethyl to isopropyl afforded 7- and fivefold improvements in potency, respectively. Moreover, the indole with the isopropyl substituent showed higher PR selectivity over GR and AR in both functional and binding assays^76^. Taken together, the important PubChem features as determined by SHAP are effective as potential PR antagonist substructures.

**Table 5:** Summary of top-twenty important features ranked by SHAP values along with their corresponding SMARTS patterns, chemical structure and substructure description.

Feature	SMARTS pattern	Substructure description
PubChemFP499	N–C:C:N	Ethylenediamine
PubChemFP335	C(~ C)(~ C)(~ C)(~ H)	2-Methylpropane
PubChemF383	C(~ S)(:C)	Ethanethiol
PubChemFP542	O–C:C–[#1]	Ethanol
PubChemFP566	O–C–C–N	2-Aminoethanol
PubChemFP652	O–C:C:C–N	3-Aminopropan-1-ol
PubChemFP382	C(~ O)(:C)(:C)	Propan-2-ol
PubChemFP338	C(~ C)(~ C)(~ H)(~ N)	Propan-2-amine
PubChemFP260	≥ 3 hetero-aromatic rings	Greater than or equal to 3 heterocyclic aromatic rings
PubChemFP569	N–C–C–N	Ethylenediamine
PubChemFP392	N(~ C)(~ C)(~ H)	N-Methylmethanamine
PubChemFP712	C–C(C)–C(C)–C	2,3-Dimethylbutane
PubChemFP420	C=O	Carbonyl group
PubChemFP717	Cc1ccc(C1)cc1	Aromatic and aliphatic carbons—(1-Ethyl-3-methylcyclopentane)
PubChemFP780	CC1CCC(C1)CC1	Aliphatic carbons (1-Ethyl-3-methylcyclopentane)
PubChemFP840	CC1CC(O)CC1	3-Methylcyclopentan-1-ol
PubChemFP590	C–C:C–O–[#1]	Propan-1-ol
PubChemFP145	≥ 1 saturated or aromatic nitrogen- containing ring size 5	Greater than or equal to 1 saturated or aromatic nitrogen-containing ring of size 5
PubChemFP430	C(–C)(–C)(=C)	2-Methylprop-1-ene
PubChemFP467	C=N–N–C	N-(Methylideneamino)methanamine

### Case study

In this section, we used molecular docking to visualize the binding interactions of compounds to PR in comparison to known modulators by using the AutoDock Vina software^66^. Out of all the compounds, the top-ten compounds with the highest docking scores indicating the highest binding affinity were selected for evaluation. As can be seen in Supplementary Fig. S2, the docking score of the top-ten compounds were − 12.67, − 12.33, − 11.82, − 11.67, − 11.48, − 11.11, − 11.10, − 11.06, − 11.03, − 11.01 kcal/mol with corresponding IC_50_ values of 0.33, 0.2, 0.025, 3.64, 0.27, 34, 4.6, 0.63, 0.63 and 19 nM, respectively. Of note, the IC_50_ values are all in the nM range highlighting that all the top-ten compounds showed strong binding affinity to PR as determined through in vitro experiments.

A further in-depth analysis of these top-ten compounds (Supplementary Fig. S2) reveals the top scaffolds pertaining to a typical steroid skeleton consisting of fused rings with three six-member rings and one five-member ring^77^ (nine out of ten compounds), nitrogen-containing compounds (nine out of ten compounds), and aliphatic compounds (all ten compounds). The compounds with the aliphatic substructures have a structural similarity not only to progesterone but also to already known PR antagonists such as asoprisnil, mifepristone and onapristone^78^. In addition, various derivatives of mifepristone such as aglepristone, lilopristone, onapristone, and telapristone have been synthesized and some are currently in clinical trials. Nine out of the top-ten compounds, resembles asoprisnil and mifepristone, which are well-known PR antagonists. Thus, the binding of PR to these compounds with high affinity is valid.

**Figure 7:**
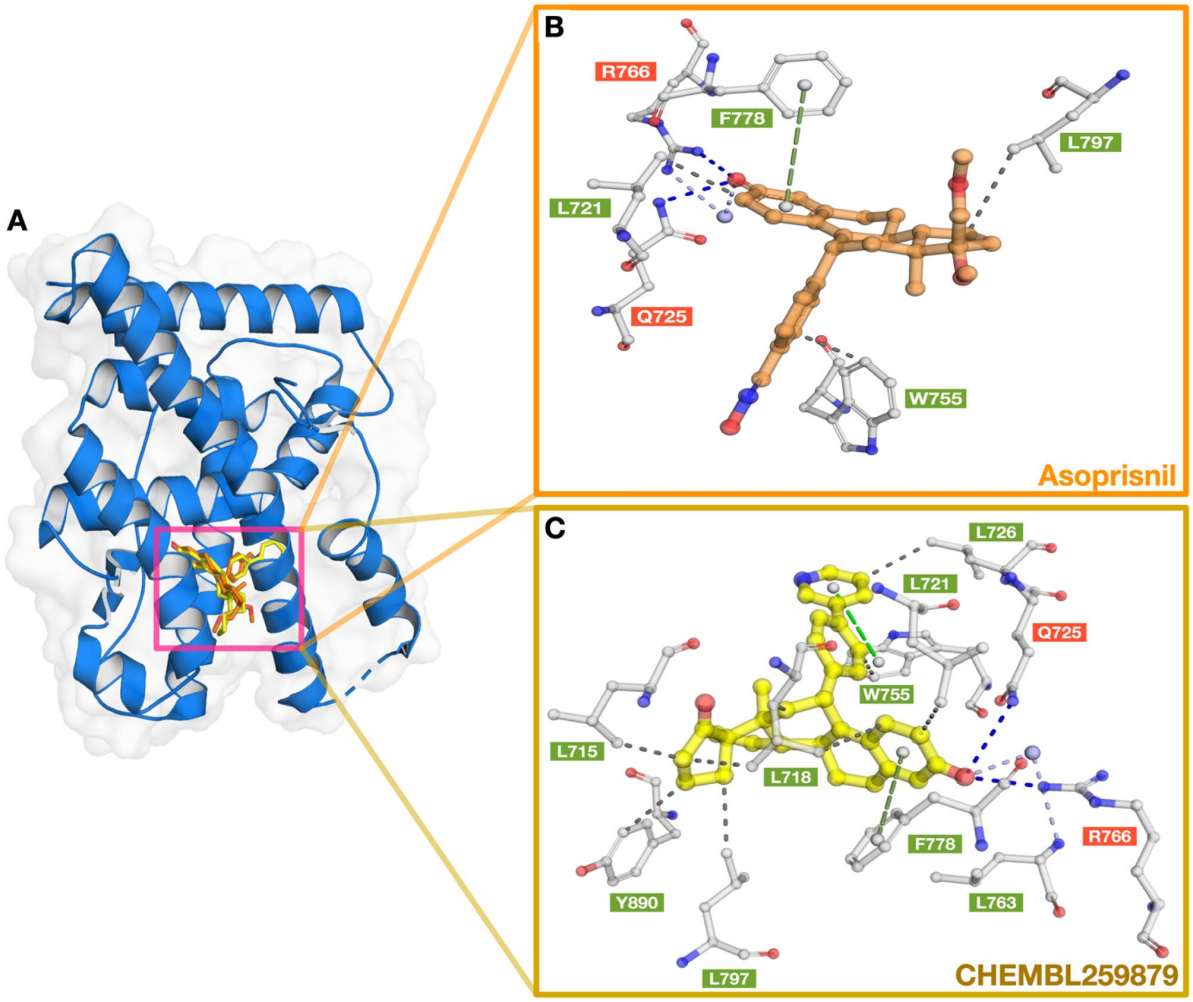
Superimposed docked pose of PR with co-crystallized ligand (PDB ID: 2OVH) (i.e., asoprisnil, **A**) and the top scoring compound as measured by AutoDock Vina (i.e., CHEMBL259879, **A**). Close-up views of the binding cavity of PR-asoprisnil (**B**) and PR-CHEMBL259879 (**C**). Hydrogen bond and hydrophobic residues are shown in red and green colored text boxes, respectively.

The docked pose of the compound with the highest docking score (i.e., CHEMBL259879; − 12.67 kcal/mol) was further investigated for its binding interactions to PR (PDB ID: 2OVH) and is portrayed in Fig. 7A and C. In addition, the binding interactions of PR (PDBID: 2OVH) with its co-crystallized structure, a SPRM (i.e., asoprisnil) in the docked pose were also elucidated using PyMOL (Fig. 7A,B). Asoprisnil exhibits antagonistic activities in endometrium, ovary, and breast tissues^79^. As observed from Fig. 7B, the docking pose between PR and asoprisnil, reveal vital interactions in the active site that consists of hydrogen bonds with NE2 and NH2 of residues Q725 and R766 at a distance of 2.91 Å and 2.74 Å, respectively, which are depicted with the blue dash lines. Additionally, residues L721, W755 and L797, were observed to form hydrophobic interactions at distances of 3.53 Å, 3.48 Å and 3.96 Å, respectively (gray dash lines). In addition, a water bridge (light purple dash lines) was shown connecting NH1 of R766 and O14 of asoprisnil. Furthermore, a perpendicular pi-stacking (depicted as a green dash line) with the phenyl ring of residue F778 was also observed at a distance of 5.32 Å. On the other hand, the docked pose of CHEMBL259879 bound to PR (Fig. 7C) reveals the interacting binding pocket residues to be comprised of Q725, and R766 forming hydrogen bonds (shown as blue dash lines) at distances of 3.66 Å, and 3.11 Å, respectively. Moreover, residues L715, L718, L721, L726, L763, W755, F778, L797, and Y890 showed binding through hydrophobic interactions that are represented with gray dash lines. In addition, two pi-pi interactions were observed, one being a staggered stacking (i.e., parallel pi-stacking, shown as a light green dash line) with W755 and the other being a pi-teeing (i.e., perpendicular pi-stacking, shown as a dark green dash line) with F778 at distances of 4.10 Å and 5.12 Å, respectively. Both these types of pi-pi interactions are known to be electrostatically attractive^80^. These results indicate that the top compound as revealed by molecular docking (i.e., CHEMBL259879) forms interactions in the binding pocket of PR that are comprised of more residues than asoprisnil. These outcomes are in accordance with SPRM binding analysis highlighting the relevance of hydrophobicity in the interactions of the ligand and catalytic residues, L718, and Q725^35^. Therefore, these interactions could be useful in determining the top substructures needed for a high-affinity PR antagonist. Additionally, competitive binding to PR was determined from the ability of compound CHEMBL259879 to exhibit antagonistic activity in a CHO (chinese hamster ovary) cell line-based assay in PubChem’s bioassay record with an IC_50_ of 0.33 nM^81,82^. Overall, these results indicate that CHEMBL259879 could be a good candidate for PR antagonism.

**Figure 8:**
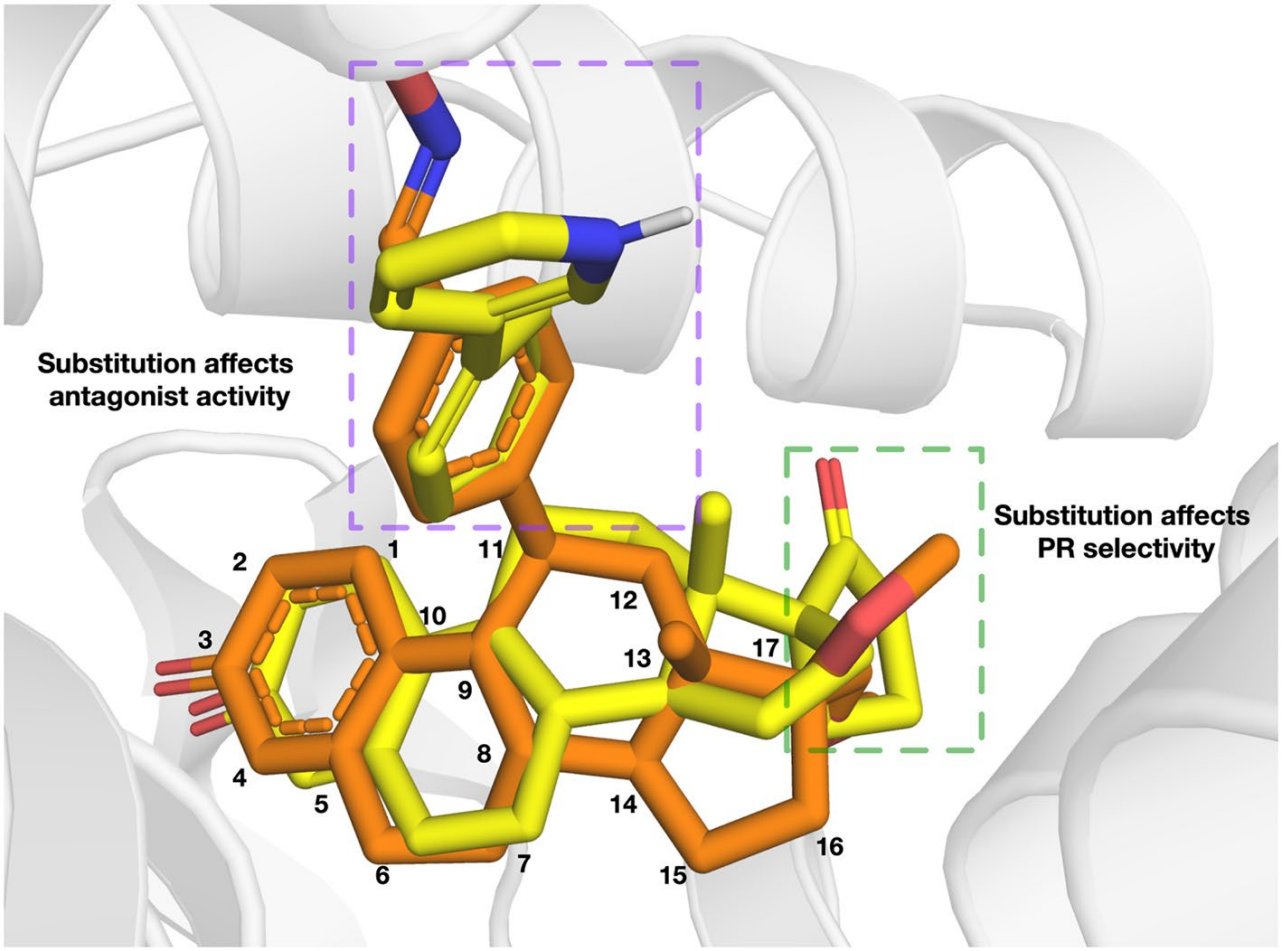
Overlaid structures of PR-Asoprisnil and PR-CHEMBL259879 where the carbons are colored orange and yellow, respectively. The C-11 and C-17 substituents are highlighted to show their effect on antagonist activity and PR selectivity, respectively.

Taking it a step further, we superimposed the ligands asoprisnil and CHEMBL259879 in the PR binding pocket to elucidate their molecular similarities and differences. As can be seen in Fig. 8, CHEMBL259879 overlays well over asoprisnil with differing scaffold substitutions at C11 and C17. A cyclopentone at C11 and a benzene with a pyridine substitution at C17 was revealed for CHEMBL259879 while asoprisnil has a benzaldehyde oxime at C11 and a dimethoxymethyl at C17. Furthermore, a key role in the binding of ligands to PR and/or GR receptor is observed to be modifications made near the C17 propinyl group (Fig. 8)^83,84^. In addition, substitutions pertaining to ethyl and pentynyl at the hydrophobic C17 produces higher PR antagonism as compared to that of asoprisnil or mifepristone^85^. Minor changes in the C17 such as a phenyl group with small, stabilizing electron-withdrawing substituents, (i.e., F, Cl, Br, and CF3), was shown to prominently increase the potency of compounds while hightening selectivity over GR^86^. As can be seen in Supplementary Fig. S2, two of the top-ten compounds (i.e., compounds (**3**) and (**10**)) contained those with the above-mentioned properties.

The substitution of 4-(dimethylamino) phenyl group at the C11 position (Fig. 8) is known to define the degree of antagonistic activity, as per the literature^83,84^. In addition, small substituents such as methyl revealed powerful agonistic properties against PR in comparison to phenyl derivatives which displayed varying degrees of antagonistic activity^83,84^. Interestingly, we can observe that nine of the top-ten compounds (i.e., compounds (**1**), (**2**), (**3**), (**4**), (**5**), (**7**), (**8**), (**9**) and (**10**)) contain substitutions of either one or two phenyl derivatives at the C11 position. Furthermore, research suggests that, nitrogen heterocycle substitution at C11 (i.e., compounds (**1**), (**4**), (**5**), (**9**) and (**10**)) especially in compounds devoid of a center of electronegativity in this region show the highest antagonistic activity^82,87^. Altogether, the top-ten compounds as determined through molecular docking, have the potential to be good candidate compounds as they have been validated for binding affinity in vitro with IC_50_ in the nM range. Furthermore, these compounds exhibit molecular substitutions which have been reported as favorable for high PR antagonism while simultaneously increasing selectivity over GR.

### StackPR web server

To guarantee that the StackPR predictor can perform the high-throughput identification of PR antagonists in a cost-effective manner, we have developed a user-friendly webserver (named StackPR) which is available at http://pmlabstack.pythonanywhere.com/StackPR. To obtain the prediction results, users are recommended to input the SMILES notation in the textbox. In addition, a step-by-step guideline on the usage of the StackPR webserver is provided at http://pmlabstack.pythonanywhere.com/about_StackPR.

## Conclusions

Breast cancer is the most detected cancer among women while gynecological cancers such as ovarian and endometrial cancers rank sixth and eighth, respectively. PR, a steroid nuclear receptor has emerged as a potential therapeutic target owing to its implications for the prognosis and development of breast cancer. Despite extensive research and high in vitro antagonistic activity of many compounds to PR, none have passed phase III clinical trials as yet. Thus, the discovery of newer PR antagonists is greatly needed. Here, we present StackPR, a novel stacked ML-based approach for the high-throughput identification of PR antagonists. We integrated six popular ML algorithms (i.e., ET, KNN, LR, PLS, RF, and SVM) coupled with twelve conventional molecular descriptors (i.e., AP2D, Circle, CKD, CKDExt, CKDGraph, Estate, FP4, FP4C, Hybrid, KR, MACCS, and PubChem) to develop the final meta-predictor using a stacking strategy. Experimental results showed that StackPR achieved impressive predictive performance with ACC, MCC, and AUC of 0.966, 0.925and 0.978, respectively on the independent test dataset. The high MCC value indicates that the proposed StackPR can effectively complement experimental studies to reduce the number of both false-positive and false-negative cases. Furthermore, analysis results based on the SHAP algorithm and molecular docking indicate that aliphatic hydrocarbons and nitrogen-containing substructures as well as substitutions at the C-11 and C-17 carbons of the steroid skeleton were the most important features for having PR antagonist activity. Moreover, non-steroidal derivatives also offer great promise particularly if potent substituents are involved. Remarkably, the docking pose of the top-scoring compound when further analyzed for binding interactions, revealed that hydrogen bonds and hydrophobic interactions were in accordance with reported PR antagonist activities. We anticipate that StackPR will be a powerful and useful computational tool for the large-scale identification of unknown PR antagonist candidates for follow-up experimental validation.

## Supplementary Information

Supplementary Information.

## Data Availability

All the data used in this study are available at http://pmlabstack.pythonanywhere.com/StackPR.
