# Supplementary material for: StackPR is a new computational approach for large-scale identification of progesterone receptor antagonists using the stacking strategy
Source: Sci Rep. 2022 Sep 30;12:16435. doi: 10.1038/s41598-022-20143-5 (PMC9525257; doi:10.1038/s41598-022-20143-5)
Supplement: Supplementary file 1 — Supplementary Information. [file 41598_2022_20143_MOESM1_ESM.docx]

**Identification of important features using GA-SAR**

The chromosome of the GA-SAR algorithm contains two main genes, binary (GA-gene) and parametric (GA-chrom). The GA-gene and GA-chrom are used for the feature selection and the parameter optimization of the ML classifier, respectively. For the mRF model, the chromosome consists of *n* = 72 binary genes (*bg*_i_) for selecting *m* important PFs and 3-bit genes for optimizing the parameters of mRF (n_estimators $\in$ {20, 50, 100, 200, 500}). If *bg_i_* = 1, the *i^th^* feature is selected to construct the mRF model; otherwise, the *i^th^* feature is excluded from the optimal feature set. The identification of informative PFs using this algorithm is described as follows:

(1) Randomly generate 50 chromosomes with randomly assigned values of binary genes to make the number of features (*m*) equal to our preferred number, where m is in the range from 5 to 20. (Initialization)

(2) Assess the prediction performances for each chromosome by performing 10-fold cross-validation test. (Evaluation)

(3) Implement a tournament selection to prepare a mating pool. (Selecting)

(4) Perform a 20-point crossover on the selected parents. (Crossover)

(5) Apply the SAR mutation operator and if the number of chosen genes are greater than the specified number of features, delete some genes. In contrast, if the number of chosen genes are less than the specified number of features, add some genes. The probability of genes to be added or deleted is referred to as SAR. (Mutation)

(6) Stop if the number of generation is equal to 50; otherwise go to Step 2. (Termination).


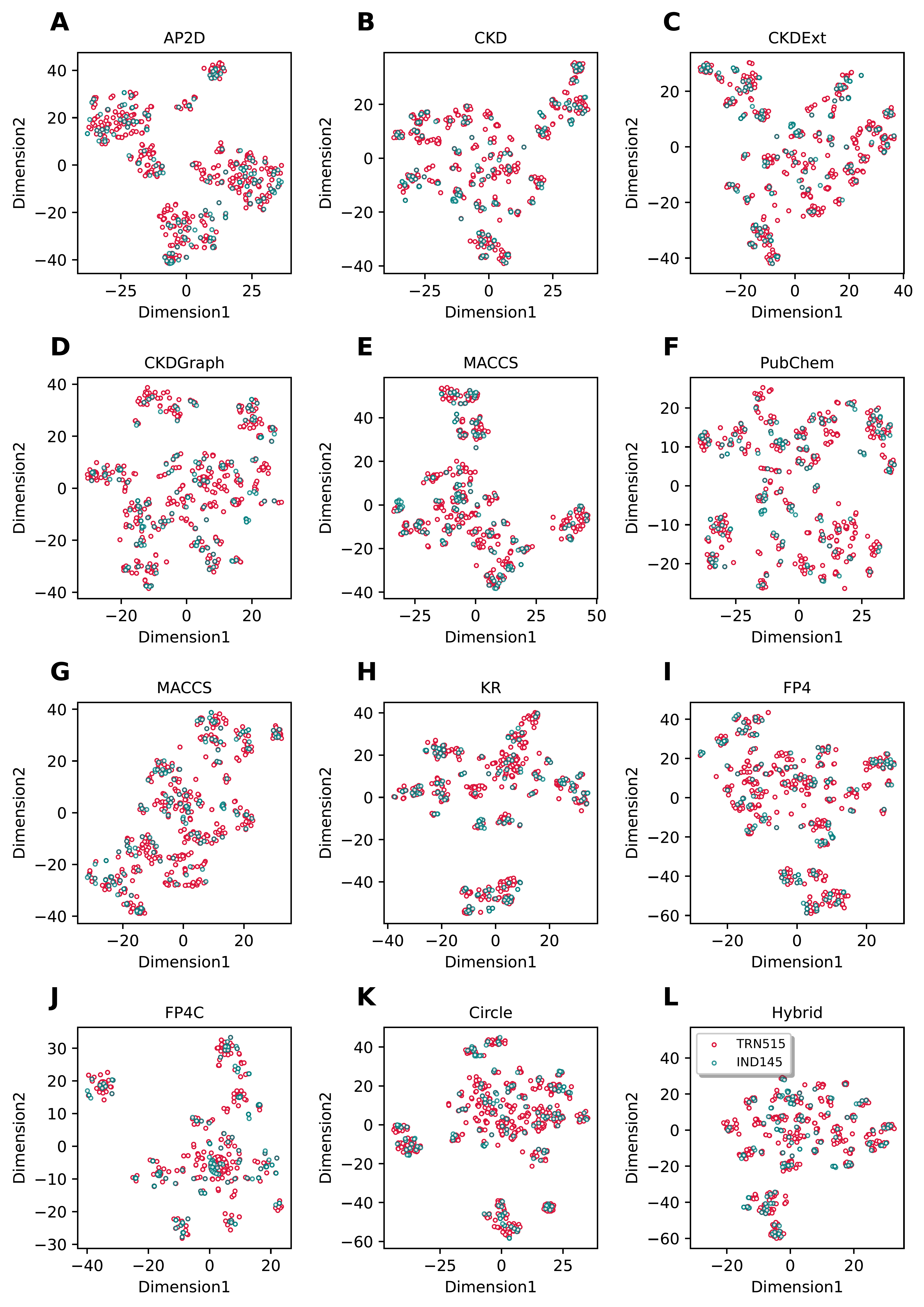


**Figure S1.** t-distributed stochastic neighbor embedding (t-SNE) distribution of the compounds in the TRN515 and IND145 datasets, where red and green spots represent compounds in TRN515 and IND145 datasets, respectively. (A–L) Distribution of the twelve fingerprint descriptors


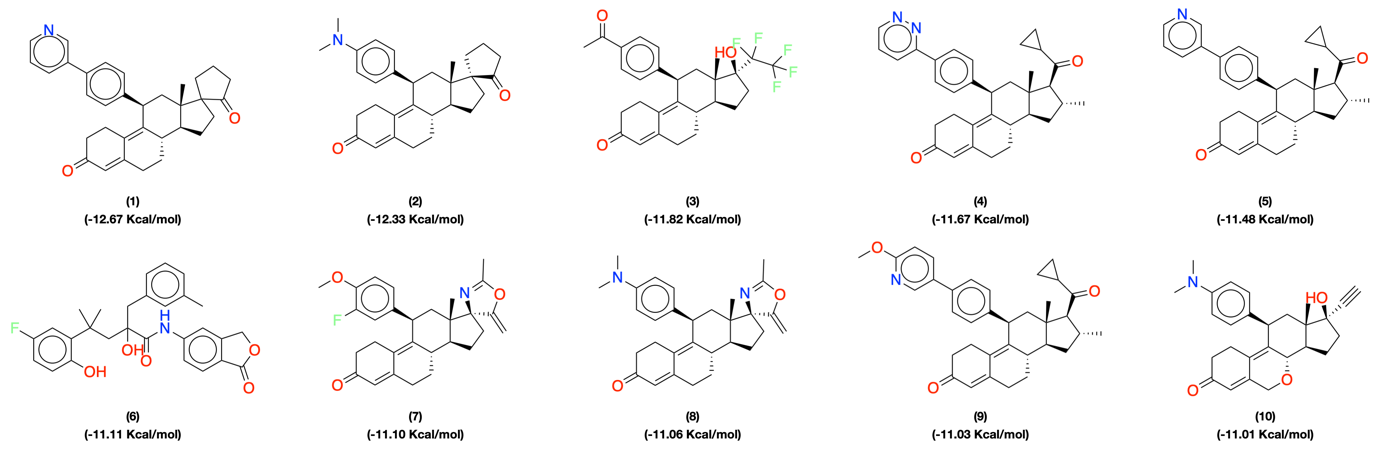


**Figure S2**. Top-ten compounds ranked by their docking scores as deduced from AutoDock Vina. The compounds are represented by numbers and their corresponding docking scores are in parentheses while their corresponding IUPAC names are in text. The set of top-ten compounds in terms of the docking score includes (8'S,11'R,13'S,14'S)-13'-methyl-11'-(4-(pyridin-3-yl)phenyl)-1',6',7',8',11',12',13',14',15',16'-decahydrospiro[cyclopentane-1,17'-cyclopenta[a]phenanthrene]-2,3'(2'H)-dione **(1)**, (1S,8'S,11'R,13'S,14'S)-11'-(4-(dimethylamino)phenyl)-13'-methyl-1',6',7',8',11',12',13',14',15',16'-decahydrospiro[cyclopentane-1,17'-cyclopenta[a]phenanthrene]-2,3'(2'H)-dione **(2),** (8S,11R,13S,14S,17S)-11-(4-acetylphenyl)-17-hydroxy-13-methyl-17-(perfluoroethyl)-1,2,6,7,8,11,12,13,14,15,16,17-dodecahydro-3H-cyclopenta[a]phenanthren-3-one **(3),** (8S,11R,13S,14S,16R,17S)-17-(cyclopropanecarbonyl)-13,16-dimethyl-11-(4-(pyridazin-3-yl)phenyl)-1,2,6,7,8,11,12,13,14,15,16,17-dodecahydro-3H-cyclopenta[a]phenanthren-3-one **(4),** (8S,11R,13S,14S,16R,17S)-17-(cyclopropanecarbonyl)-13,16-dimethyl-11-(4-(pyridin-3-yl)phenyl)-1,2,6,7,8,11,12,13,14,15,16,17-dodecahydro-3H-cyclopenta[a]phenanthren-3-one **(5),** 4-(5-fluoro-2-hydroxyphenyl)-2-hydroxy-4-methyl-2-(3-methylbenzyl)-N-(1-oxo-1,3-dihydroisobenzofuran-5-yl)pentanamide **(6),** (8S,11R,13S,14S,17S)-11-(3-fluoro-4-methoxyphenyl)-2',13-dimethyl-5'-methylene-1,6,7,8,11,12,13,14,15,16-decahydro-5'H-spiro[cyclopenta[a]phenanthrene-17,4'-oxazol]-3(2H)-one **(7),** (8S,11R,13S,14S,17S)-11-(4-(dimethylamino)phenyl)-2',13-dimethyl-5'-methylene-1,6,7,8,11,12,13,14,15,16-decahydro-5'H-spiro[cyclopenta[a]phenanthrene-17,4'-oxazol]-3(2H)-one **(8),** (8S,11R,13S,14S,16R,17S)-17-(cyclopropanecarbonyl)-11-(4-(6-methoxypyridin-3-yl)phenyl)-13,16-dimethyl-1,2,6,7,8,11,12,13,14,15,16,17-dodecahydro-3H-cyclopenta[a]phenanthren-3-one **(9),** and (1R,3aR,3bS,10R,11aS)-10-(4-(dimethylamino)phenyl)-1-ethynyl-1-hydroxy-11a-methyl-1,2,3,3a,3b,8,9,10,11,11a-decahydrobenzo[c]cyclopenta[h]chromen-7(5H)-one **(10)**

**Table S1** Cross-validation results of 72 baseline models developed using six different ML algorithms and twelve molecular descriptors.

| **Descriptor** | **ML** | **ACC** | **Sn** | **Sp** | **MCC** | **AUC** |
| --- | --- | --- | --- | --- | --- | --- |
| AP2D | ET | 0.879 | 0.924 | 0.798 | 0.734 | 0.926 |
|  | KNN | 0.844 | 0.946 | 0.663 | 0.656 | 0.805 |
|  | LR | 0.874 | 0.927 | 0.779 | 0.722 | 0.925 |
|  | PLS | 0.811 | 0.878 | 0.692 | 0.584 | 0.885 |
|  | RF | 0.881 | 0.935 | 0.784 | 0.737 | 0.936 |
|  | SVM | 0.858 | 0.916 | 0.755 | 0.687 | 0.918 |
| Circle | ET | 0.931 | 0.943 | 0.909 | 0.850 | 0.973 |
|  | KNN | 0.915 | 0.943 | 0.865 | 0.815 | 0.904 |
|  | LR | 0.927 | 0.946 | 0.894 | 0.842 | 0.970 |
|  | PLS | 0.910 | 0.914 | 0.904 | 0.808 | 0.964 |
|  | RF | 0.929 | 0.943 | 0.904 | 0.846 | 0.974 |
|  | SVM | 0.927 | 0.935 | 0.913 | 0.843 | 0.970 |
| CKD | ET | 0.898 | 0.930 | 0.841 | 0.777 | 0.963 |
|  | KNN | 0.903 | 0.954 | 0.813 | 0.788 | 0.883 |
|  | LR | 0.927 | 0.957 | 0.875 | 0.841 | 0.963 |
|  | PLS | 0.896 | 0.916 | 0.861 | 0.775 | 0.957 |
|  | RF | 0.907 | 0.941 | 0.846 | 0.796 | 0.967 |
|  | SVM | 0.910 | 0.932 | 0.870 | 0.804 | 0.960 |
| CKDExt | ET | 0.912 | 0.941 | 0.861 | 0.807 | 0.957 |
|  | KNN | 0.905 | 0.954 | 0.817 | 0.791 | 0.886 |
|  | LR | 0.913 | 0.954 | 0.841 | 0.811 | 0.962 |
|  | PLS | 0.894 | 0.914 | 0.861 | 0.772 | 0.960 |
|  | RF | 0.908 | 0.943 | 0.846 | 0.799 | 0.969 |
|  | SVM | 0.905 | 0.924 | 0.870 | 0.794 | 0.962 |
| CKDGraph | ET | 0.872 | 0.908 | 0.808 | 0.721 | 0.923 |
|  | KNN | 0.851 | 0.932 | 0.707 | 0.671 | 0.820 |
|  | LR | 0.869 | 0.941 | 0.740 | 0.710 | 0.904 |
|  | PLS | 0.815 | 0.946 | 0.580 | 0.588 | 0.865 |
|  | RF | 0.882 | 0.935 | 0.788 | 0.741 | 0.934 |
|  | SVM | 0.855 | 0.935 | 0.712 | 0.679 | 0.904 |
| Estate | ET | 0.891 | 0.924 | 0.832 | 0.762 | 0.938 |
|  | KNN | 0.879 | 0.946 | 0.760 | 0.734 | 0.853 |
|  | LR | 0.785 | 0.873 | 0.630 | 0.523 | 0.870 |
|  | PLS | 0.799 | 0.876 | 0.663 | 0.555 | 0.865 |
|  | RF | 0.888 | 0.930 | 0.813 | 0.753 | 0.950 |
|  | SVM | 0.865 | 0.924 | 0.760 | 0.703 | 0.927 |
| FP4 | ET | 0.912 | 0.943 | 0.856 | 0.807 | 0.942 |
|  | KNN | 0.896 | 0.954 | 0.793 | 0.772 | 0.874 |
|  | LR | 0.875 | 0.916 | 0.803 | 0.727 | 0.930 |
|  | PLS | 0.869 | 0.892 | 0.827 | 0.716 | 0.918 |
|  | RF | 0.910 | 0.949 | 0.841 | 0.803 | 0.956 |
|  | SVM | 0.891 | 0.919 | 0.841 | 0.763 | 0.948 |
| FP4C | ET | 0.803 | 0.849 | 0.721 | 0.571 | 0.861 |
|  | KNN | 0.753 | 0.976 | 0.356 | 0.454 | 0.666 |
|  | LR | 0.770 | 0.868 | 0.596 | 0.486 | 0.833 |
|  | PLS | 0.746 | 0.862 | 0.538 | 0.427 | 0.824 |
|  | RF | 0.799 | 0.865 | 0.683 | 0.558 | 0.861 |
|  | SVM | 0.789 | 0.862 | 0.659 | 0.534 | 0.836 |
| Hybrid | ET | 0.901 | 0.935 | 0.841 | 0.784 | 0.958 |
|  | KNN | 0.900 | 0.951 | 0.808 | 0.780 | 0.880 |
|  | LR | 0.903 | 0.943 | 0.832 | 0.788 | 0.953 |
|  | PLS | 0.896 | 0.941 | 0.817 | 0.772 | 0.956 |
|  | RF | 0.896 | 0.930 | 0.837 | 0.773 | 0.967 |
|  | SVM | 0.912 | 0.938 | 0.865 | 0.808 | 0.963 |
| KR | ET | 0.927 | 0.959 | 0.870 | 0.841 | 0.970 |
|  | KNN | 0.903 | 0.949 | 0.822 | 0.788 | 0.885 |
|  | LR | 0.908 | 0.935 | 0.861 | 0.800 | 0.952 |
|  | PLS | 0.898 | 0.932 | 0.837 | 0.777 | 0.947 |
|  | RF | 0.915 | 0.957 | 0.841 | 0.814 | 0.969 |
|  | SVM | 0.903 | 0.924 | 0.865 | 0.790 | 0.958 |
| MACCS | ET | 0.913 | 0.941 | 0.865 | 0.811 | 0.960 |
|  | KNN | 0.910 | 0.954 | 0.832 | 0.803 | 0.893 |
|  | LR | 0.888 | 0.927 | 0.817 | 0.754 | 0.945 |
|  | PLS | 0.836 | 0.884 | 0.750 | 0.640 | 0.916 |
|  | RF | 0.912 | 0.949 | 0.846 | 0.807 | 0.963 |
|  | SVM | 0.896 | 0.924 | 0.846 | 0.774 | 0.949 |
| PubChem | ET | 0.894 | 0.927 | 0.837 | 0.770 | 0.951 |
|  | KNN | 0.910 | 0.954 | 0.832 | 0.803 | 0.893 |
|  | LR | 0.888 | 0.935 | 0.803 | 0.753 | 0.935 |
|  | PLS | 0.875 | 0.919 | 0.798 | 0.727 | 0.940 |
|  | RF | 0.901 | 0.927 | 0.856 | 0.785 | 0.959 |
|  | SVM | 0.889 | 0.922 | 0.832 | 0.758 | 0.954 |

**Table S2** Independent test results of 72 baseline models developed using six different ML algorithms and twelve molecular descriptors.

| **Descriptor** | **ML** | **ACC** | **Sn** | **Sp** | **MCC** | **AUC** |
| --- | --- | --- | --- | --- | --- | --- |
| AP2D | ET | 0.897 | 0.925 | 0.846 | 0.774 | 0.952 |
|  | KNN | 0.883 | 0.968 | 0.731 | 0.744 | 0.849 |
|  | LR | 0.862 | 0.882 | 0.827 | 0.703 | 0.913 |
|  | PLS | 0.800 | 0.882 | 0.654 | 0.555 | 0.885 |
|  | RF | 0.897 | 0.946 | 0.808 | 0.773 | 0.965 |
|  | SVM | 0.848 | 0.903 | 0.750 | 0.666 | 0.912 |
| Circle | ET | 0.945 | 0.946 | 0.942 | 0.881 | 0.983 |
|  | KNN | 0.945 | 0.968 | 0.904 | 0.879 | 0.936 |
|  | LR | 0.931 | 0.957 | 0.885 | 0.849 | 0.981 |
|  | PLS | 0.897 | 0.882 | 0.923 | 0.786 | 0.952 |
|  | RF | 0.945 | 0.946 | 0.942 | 0.881 | 0.983 |
|  | SVM | 0.938 | 0.946 | 0.923 | 0.866 | 0.983 |
| CKD | ET | 0.910 | 0.935 | 0.865 | 0.804 | 0.956 |
|  | KNN | 0.903 | 0.935 | 0.846 | 0.789 | 0.891 |
|  | LR | 0.931 | 0.946 | 0.904 | 0.850 | 0.978 |
|  | PLS | 0.897 | 0.903 | 0.885 | 0.779 | 0.949 |
|  | RF | 0.903 | 0.935 | 0.846 | 0.789 | 0.974 |
|  | SVM | 0.931 | 0.957 | 0.885 | 0.849 | 0.973 |
| CKDExt | ET | 0.910 | 0.935 | 0.865 | 0.804 | 0.964 |
|  | KNN | 0.897 | 0.935 | 0.827 | 0.773 | 0.881 |
|  | LR | 0.938 | 0.957 | 0.904 | 0.865 | 0.981 |
|  | PLS | 0.883 | 0.903 | 0.846 | 0.746 | 0.947 |
|  | RF | 0.910 | 0.935 | 0.865 | 0.804 | 0.967 |
|  | SVM | 0.938 | 0.968 | 0.885 | 0.864 | 0.974 |
| CKDGraph | ET | 0.890 | 0.914 | 0.846 | 0.760 | 0.941 |
|  | KNN | 0.855 | 0.946 | 0.692 | 0.680 | 0.819 |
|  | LR | 0.876 | 0.925 | 0.788 | 0.727 | 0.923 |
|  | PLS | 0.779 | 0.914 | 0.538 | 0.502 | 0.837 |
|  | RF | 0.869 | 0.903 | 0.808 | 0.714 | 0.934 |
|  | SVM | 0.848 | 0.946 | 0.673 | 0.665 | 0.923 |
| Estate | ET | 0.890 | 0.925 | 0.827 | 0.758 | 0.915 |
|  | KNN | 0.841 | 0.946 | 0.654 | 0.649 | 0.800 |
|  | LR | 0.724 | 0.806 | 0.577 | 0.391 | 0.793 |
|  | PLS | 0.697 | 0.785 | 0.538 | 0.330 | 0.781 |
|  | RF | 0.876 | 0.925 | 0.788 | 0.727 | 0.934 |
|  | SVM | 0.800 | 0.860 | 0.692 | 0.560 | 0.879 |
| FP4 | ET | 0.890 | 0.925 | 0.827 | 0.758 | 0.918 |
|  | KNN | 0.848 | 0.957 | 0.654 | 0.666 | 0.805 |
|  | LR | 0.841 | 0.903 | 0.731 | 0.650 | 0.907 |
|  | PLS | 0.834 | 0.828 | 0.846 | 0.656 | 0.904 |
|  | RF | 0.897 | 0.935 | 0.827 | 0.773 | 0.945 |
|  | SVM | 0.890 | 0.914 | 0.846 | 0.760 | 0.934 |
| FP4C | ET | 0.862 | 0.925 | 0.750 | 0.695 | 0.923 |
|  | KNN | 0.759 | 0.978 | 0.365 | 0.469 | 0.672 |
|  | LR | 0.814 | 0.839 | 0.769 | 0.601 | 0.852 |
|  | PLS | 0.800 | 0.882 | 0.654 | 0.555 | 0.843 |
|  | RF | 0.855 | 0.925 | 0.731 | 0.680 | 0.919 |
|  | SVM | 0.855 | 0.925 | 0.731 | 0.680 | 0.905 |
| Hybrid | ET | 0.910 | 0.925 | 0.885 | 0.806 | 0.969 |
|  | KNN | 0.903 | 0.935 | 0.846 | 0.789 | 0.891 |
|  | LR | 0.917 | 0.946 | 0.865 | 0.819 | 0.971 |
|  | PLS | 0.883 | 0.925 | 0.808 | 0.743 | 0.933 |
|  | RF | 0.903 | 0.925 | 0.865 | 0.790 | 0.969 |
|  | SVM | 0.903 | 0.925 | 0.865 | 0.790 | 0.961 |
| KR | ET | 0.917 | 0.925 | 0.904 | 0.822 | 0.972 |
|  | KNN | 0.924 | 0.957 | 0.865 | 0.834 | 0.911 |
|  | LR | 0.897 | 0.935 | 0.827 | 0.773 | 0.963 |
|  | PLS | 0.910 | 0.903 | 0.923 | 0.811 | 0.953 |
|  | RF | 0.917 | 0.935 | 0.885 | 0.820 | 0.970 |
|  | SVM | 0.910 | 0.914 | 0.904 | 0.808 | 0.954 |
| MACCS | ET | 0.917 | 0.957 | 0.846 | 0.819 | 0.955 |
|  | KNN | 0.897 | 0.946 | 0.808 | 0.773 | 0.877 |
|  | LR | 0.897 | 0.968 | 0.769 | 0.774 | 0.938 |
|  | PLS | 0.841 | 0.860 | 0.808 | 0.660 | 0.897 |
|  | RF | 0.924 | 0.957 | 0.865 | 0.834 | 0.955 |
|  | SVM | 0.890 | 0.925 | 0.827 | 0.758 | 0.949 |
| PubChem | ET | 0.924 | 0.935 | 0.904 | 0.836 | 0.948 |
|  | KNN | 0.883 | 0.935 | 0.788 | 0.742 | 0.862 |
|  | LR | 0.910 | 0.946 | 0.846 | 0.803 | 0.942 |
|  | PLS | 0.841 | 0.882 | 0.769 | 0.654 | 0.910 |
|  | RF | 0.931 | 0.946 | 0.904 | 0.850 | 0.955 |
|  | SVM | 0.897 | 0.925 | 0.846 | 0.774 | 0.938 |

**Table S3** Average (±standard deviations) cross-validation performance of each molecular descriptor over six different ML algorithms.

| **Descriptor** | **ACC** | **Sn** | **Sp** | **MCC** | **AUC** |
| --- | --- | --- | --- | --- | --- |
| AP2D | 0.858±0.027 | 0.921±0.023 | 0.745±0.055 | 0.687±0.059 | 0.899±0.049 |
| Circle | 0.923±0.009 | 0.937±0.012 | 0.898±0.017 | 0.834±0.018 | 0.959±0.027 |
| CKD | 0.907±0.011 | 0.938±0.016 | 0.851±0.023 | 0.797±0.024 | 0.949±0.032 |
| CKDExt | 0.906±0.007 | 0.938±0.016 | 0.849±0.019 | 0.796±0.014 | 0.949±0.031 |
| CKDGraph | 0.857±0.024 | 0.933±0.013 | 0.723±0.081 | 0.685±0.054 | 0.892±0.042 |
| Estate | 0.851±0.047 | 0.912±0.030 | 0.743±0.081 | 0.672±0.105 | 0.901±0.042 |
| FP4 | 0.892±0.018 | 0.929±0.024 | 0.827±0.024 | 0.765±0.038 | 0.928±0.030 |
| FP4C | 0.777±0.024 | 0.880±0.047 | 0.592±0.133 | 0.505±0.058 | 0.814±0.074 |
| Hybrid | 0.901±0.006 | 0.940±0.007 | 0.833±0.020 | 0.784±0.013 | 0.946±0.033 |
| KR | 0.909±0.011 | 0.943±0.014 | 0.849±0.019 | 0.802±0.023 | 0.947±0.032 |
| MACCS | 0.893±0.029 | 0.930±0.025 | 0.826±0.041 | 0.765±0.065 | 0.938±0.028 |
| PubChem | 0.893±0.012 | 0.931±0.013 | 0.826±0.022 | 0.766±0.026 | 0.939±0.024 |

**Table S4** Average (±standard deviations) cross-validation performance of each ML over twelve molecular descriptors.

| **Method** | **ACC** | **Sn** | **Sp** | **MCC** | **AUC** |
| --- | --- | --- | --- | --- | --- |
| ET | 0.894±0.034 | 0.927±0.028 | 0.837±0.047 | 0.770±0.074 | 0.944±0.031 |
| KNN | 0.881±0.046 | 0.951±0.010 | 0.756±0.138 | 0.738±0.103 | 0.854±0.066 |
| LR | 0.877±0.051 | 0.927±0.029 | 0.789±0.093 | 0.730±0.114 | 0.929±0.041 |
| PLS | 0.854±0.052 | 0.906±0.027 | 0.761±0.118 | 0.678±0.118 | 0.916±0.046 |
| RF | 0.894±0.033 | 0.934±0.023 | 0.824±0.054 | 0.768±0.073 | 0.950±0.031 |
| SVM | 0.883±0.037 | 0.921±0.020 | 0.816±0.076 | 0.745±0.084 | 0.937±0.038 |

**Table S5** Performance comparison of StackPR and top five baseline models on the training and independent test datasets.

| **Evaluation strategy** | **Method** | **TP** | **FP** | **TN** | **FN** |
| --- | --- | --- | --- | --- | --- |
| Cross-validation | ET-Circle | 290 | 18 | 190 | 17 |
|  | RF-Circle | 290 | 19 | 189 | 17 |
|  | SVM-Circle | 288 | 18 | 190 | 19 |
|  | LR-Circle | 291 | 22 | 186 | 16 |
|  | LR-CKD | 295 | 26 | 182 | 12 |
|  | StackPR | 299 | 18 | 190 | 8 |
| Independent test | ET-Circle | 88 | 3 | 49 | 5 |
|  | RF-Circle | 88 | 3 | 49 | 5 |
|  | SVM-Circle | 88 | 4 | 48 | 5 |
|  | LR-Circle | 89 | 6 | 46 | 4 |
|  | LR-CKD | 87 | 4 | 48 | 6 |
|  | StackPR | 91 | 3 | 49 | 2 |

**Table S6.** Cross-validation results of RF classifiers trained with different feature encodings on the training dataset.

| **Feature** | **ACC** | **Sn** | **Sp** | **MCC** | **AUC** |
| --- | --- | --- | --- | --- | --- |
| AP2D | 0.881 | 0.935 | 0.784 | 0.737 | 0.936 |
| Circle | 0.929 | 0.943 | 0.904 | 0.846 | 0.974 |
| CKD | 0.907 | 0.941 | 0.846 | 0.796 | 0.967 |
| CKDExt | 0.908 | 0.943 | 0.846 | 0.799 | 0.969 |
| CKDGraph | 0.882 | 0.935 | 0.788 | 0.741 | 0.934 |
| Estate | 0.888 | 0.930 | 0.813 | 0.753 | 0.950 |
| FP4 | 0.910 | 0.949 | 0.841 | 0.803 | 0.956 |
| FP4C | 0.799 | 0.865 | 0.683 | 0.558 | 0.861 |
| Hybrid | 0.896 | 0.930 | 0.837 | 0.773 | 0.967 |
| KR | 0.915 | 0.957 | 0.841 | 0.814 | 0.969 |
| MACCS | 0.912 | 0.949 | 0.846 | 0.807 | 0.963 |
| PubChem | 0.901 | 0.927 | 0.856 | 0.785 | 0.959 |
| PF (Our feature) | 0.950 | 0.970 | 0.913 | 0.893 | 0.976 |

**Table S7.** Independent test results of RF classifiers trained with different feature encodings on the independent test dataset.

| **Feature** | **ACC** | **Sn** | **Sp** | **MCC** | **AUC** |
| --- | --- | --- | --- | --- | --- |
| AP2D | 0.897 | 0.946 | 0.808 | 0.773 | 0.965 |
| Circle | 0.945 | 0.946 | 0.942 | 0.881 | 0.983 |
| CKD | 0.903 | 0.935 | 0.846 | 0.789 | 0.974 |
| CKDExt | 0.910 | 0.935 | 0.865 | 0.804 | 0.967 |
| CKDGraph | 0.869 | 0.903 | 0.808 | 0.714 | 0.934 |
| Estate | 0.876 | 0.925 | 0.788 | 0.727 | 0.934 |
| FP4 | 0.897 | 0.935 | 0.827 | 0.773 | 0.945 |
| FP4C | 0.855 | 0.925 | 0.731 | 0.680 | 0.919 |
| Hybrid | 0.903 | 0.925 | 0.865 | 0.790 | 0.969 |
| KR | 0.917 | 0.935 | 0.885 | 0.820 | 0.970 |
| MACCS | 0.924 | 0.957 | 0.865 | 0.834 | 0.955 |
| PubChem | 0.931 | 0.946 | 0.904 | 0.850 | 0.955 |
| PF (Our feature) | 0.966 | 0.978 | 0.942 | 0.925 | 0.978 |
